# Polymorphisms in Promoter Region of the Interferon-Gamma Receptor-1 Gene and its Relation with Susceptibility to Brucellosis

**DOI:** 10.30699/ijp.2019.91536.1888

**Published:** 2019-08-01

**Authors:** Zahra Naseri, Nasrin Bahmani, Mohammad Yosef Alikhani, Seyed Hamid Hashemi, Ghodratollah Roshanaei

**Affiliations:** 1 Blood Transfusion Research Center, High Institute for Research and Education in Transfusion, Hamadan, Iran; 2 Zoonoses Research Center, Research Institute for Health Development, Kurdistan University of Medical Sciences, Sanandaj, Iran; 3 Department of Microbiology, Hamadan University of Medical Sciences, Hamadan, Iran; 4 Brucellosis Research Center, Hamadan University of Medical Sciences, Hamadan, Iran; 5 Department of Biostatistics and Epidemiology, Hamadan University of Health Sciences, Hamadan, Iran

**Keywords:** Brucella infection, Interferon -gamma receptor, DNA restriction enzyme, Single nucleotide polymorphism

## Abstract

**Background & Objective:**

Brucellosis is one of the most prevalent bacterial zoonotic diseases which afflicts both humans and animals. Genetic factors play an important role in susceptibility to brucellosis. One of these factors is interferon-gamma (IFN-), which is vital in the defense mechanism against infectious diseases such as brucellosis. The purpose of this study was to evaluate the relationship between two single nucleotide polymorphisms (SNPs) at positions -611 and -56 within the promoter region of interferon-gamma receptor-1 gene (IFN- R1) and brucellosis.

**Methods:**

In this research, the genomic DNA was collected from 60 peripheral blood samples infected with brucellosis and 68 healthy volunteers. DNA was extracted by salting out method. Then, DNA genotypes were analyzed using polymerase chain reaction-restriction fragment length polymorphisms (PCR-RFLP).

**Results:**

The results showed that there is a significant difference in -611 SNP frequencies between control and patient groups. At position -611, CC genotype was related to patient group (*P*=0.024) and TT genotype was related to the control group. According to the results, males had a higher frequency of *Brucella* infection.

**Conclusion:**

The presence of C allele in position -611 in IFNγ R1 gene promoter was related to a higher risk of disease and susceptibility to brucellosis. Moreover, the presence of T allele in position γ

## Introduction

Brucellosis is the most common bacterial zoonotic disease across the world, which afflicts annually more than half a million people. Despite many efforts made to control the spread, this organism is still a major health problem in many parts of the world such as the Mediterranean region, western Asia, Africa, and America. It also remains to be an endemic in some regions such as Iran, Turkey, and the Arabian Peninsula (1). Brucellosis is caused by the *Brucella* genus; and it can be transmitted by the consumption of contaminated milk and dairy products, vaginal secretion, respiratory tract, and ingestion of the infected meat of animals (2). It has been proven that effective immunity against intracellular pathogen cells such as *Brucella* requires cellular immunity by stimulation. Th1 responses are essential for eliminating the *Brucella* infection (3).

INF-γ is one of the main cytokines in Th1 responses, which plays a major role in the anti-*Brucella* action, and it is also the main cause of immunity against *Brucella* spp. (4). Interferon-gamma receptor (IFN-γ R) is a heterodimeric receptor consisting of two chains (IFN-γ R1, IFN-γ R2). IFN-γ R1 binds to the ligand and IFN-γ R2 is involved in signaling a pathway on macrophages (5). Regarding the relationship between single nucleotide polymorphism (SNP) and susceptibility to brucellosis, it has been indicated that the development of brucellosis and acute or chronic diseases can help create new therapeutic strategies for brucellosis patients. Numerous evidences suggest that SNPs within the binding site of transcription factors may reduce the function of the promoter of a cytokine/receptor complex, and can affect the symptoms of the disease (6).

Allelic variation can affect protein production at multiple loci SNPs as a response to pathogens. Therefore, it predisposes people to accept or reject diseases. An SNP array is a useful tool to study minor variations and SNP-based genetic analysis, which can be used to determine susceptibility genes as well as determine the map disease (7). Several polymorphisms in the gene of IFN-γ have been reported including +G5644A, -G183T, +A874T, +C764G, +G3810A, and +A2109G loci to influence clinical features, susceptibility, and the development of the diseases (8).

In this study, we selected two functional polymorphism sites of the IFN-γ gene that had been previously reported. One polymorphism in the promoter region of IFNγ R1 was located at position -611 and another one at position -56 in brucellosis patients compared to controls (9). Demographic characteristics were also determined in the samples.

## Materials and Methods

**Study Groups**

A total of 128 blood samples were taken from two patient and control groups, including 60 hospitalized patients with brucellosis at the Sina hospital of Hamadan, Iran and 68 healthy volunteers from May 2016 until April 2017. The diagnosis of brucellosis was based on clinical symptoms (fever, arthralgia, malaise, night sweating, weakness, hepatomegaly, and splenomegaly) and the existence of high titers of specific antibodies. Titers were set at 1.160 for Sero-agglutination test (SAT) or 1.320 for Coombs anti-*Brucella* test on 60 patients with infected brucellosis (10). The control group, which was randomly selected, did not show any clinical symptoms after 6 months of follow up. The patient and control groups were from the same geographical areas. The blood samples were collected in sterile tubes using ethylenediaminetetraacetic acid (EDTA), transferred to the laboratory, and kept at -20°C.

DNA Extraction

Genomic DNA was extracted from EDTA anticoagulated venous blood using the salting out method (11). Briefly, 0.5 ml of blood samples with 1 ml of erythrocyte lyses solution (320 mM saccharose, 1% Triton X-100, 5 mM MgCl2, 10 mM Tris HCl [pH 7.5]) were mixed and centrifuged at 15000×g for 2 min. This step was repeated three times. 400 microliters of lyses buffer (10 mM Sodium Acetate [pH 8], 10 mM Tris-Hcl‚ 10mM EDTA‚ 1% SDS) and proteinase K (10 mg/ml) were added to the pellet and mixed. After incubation at 55°C for 20 min, 100 µL of ammonium acetate (7.5 M) was added and centrifuged for 10 min. Finally, 200 µL of absolute ethanol was added to the supernatant and after centrifuging, the pellets were kept in 50 μL of TE buffer and stored at 4°C for PCR or at -20°C for long-term storage.

PCR Assay

PCR was carried out at a final volume as much as a mixture of 25 µl containing 12.5 µl of mastermix (Fermentas Co), 2 µl total DNA extracted, 1 µl of each primer and distilled water; and the mixture was processed in a thermocycler (Eppendorf Co). PCR conditions for -611 and -56 SNP are shown in Tables 1 and 2, respectively. PCR products were digested by Hpy188I restriction enzyme (New England Biolab) for -611 SNP and affair restriction enzyme for -56 SNP (New England Biolab). For PCR-RFLP, 15 μl reactions were prepared. Restriction enzymes were used according to manufacturer’s instruction. Briefly, 5 μl of PCR product, 1 μl of enzyme buffer, 0.5 μl of restriction enzyme, and sterile distilled water up to 15 μl were used. The micro-tubes were incubated for four hours at 37°C. The digested products were isolated on 1.5% agarose and visualized under UV light. The primers and restriction enzymes used are listed in Table 3.

**Table 1:** PCR conditions were optimized for -611 SNP as follows

Cycle	Time	Tm C^◦^	Steps
	5 min	95	Predenaturation
35	35 s	95	Denaturation
	35 s	61	Annealing
	60 s	72	Extension
	10 min	72	Final extension

**Table 2:** PCR conditions were optimized for -56 SNP as follows

Cycle	Time	Tm C^◦^	Steps
	5 min	95	Predenaturation
35	30 s	94	Denaturation
	30s	59	Annealing
	60 s	72	Extension
	10 min	72	Final extension

**Table 3:** Primers and restriction enzymes used for genotyping *Brucella*

SNP, position	Restriction Enzyme	PCR primer sequence (5′- 3′)	Allele Phenotype
-611T/C(P)	Hpy188I	F:CTCTTCATGAGAGGCTGTCT R:TAACTCTTGGAGTTCACCTGG	T: 260bp C:240 + 20bp
-56A/G(P)	AfeI	F:TGCATGACAAGGGGTAGGAG R:CAACCAGGTGAAGTCCAAGAG	A: 430bp G:339 + 91bp

Statistical Analysis

 The data was analyzed using SPSS version 16 and statistical tests, including independent Pearson’s chi-square and adjusted logistic regression models at 95% confidence interval (CI) and odds ratio (OR) (P-value<0.05). According to statistical analysis, there could be three genotypes for -611 (TT, TC, and CC) and three for -56 (AA, GA, and GG).

## Results

According to statistical analysis, the demographic characteristics of patient and control groups are presented in Table 4. The logistic regression and independent Pearson’s chi-square showed that there is no significant relationship between the brucellosis and age (*P*>0.05), aspartate aminotransferase (AST) (*P*>0.9), alanine aminotransferase (ALT) (*P*>0.8), and alkaline phosphatase (ALP) (*P*>0.76) (Data are not shown). In addition, the statistical analysis showed that there is a significant correlation between the brucellosis and gender (*P*<0.02), education level (*P*<0.014), history of brucellosis (*P*<0.014), geographical location (*P*<0.001), and domestication (*P*<0.001). 

**Figure 1:**
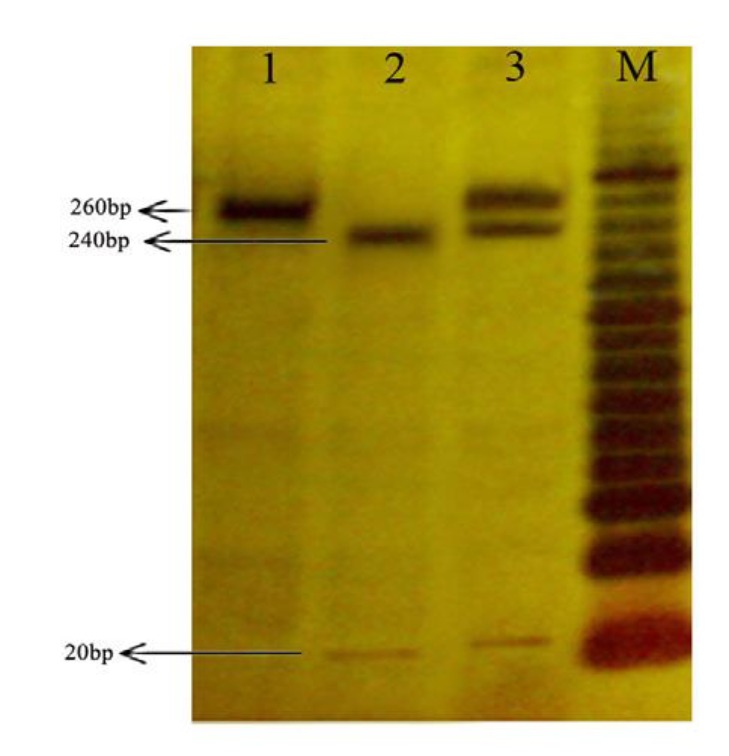
The digestion pattern of Hpy188I restriction enzyme on 18% polyacrylamide gel at -611 SNP. M marker 20bp, 1 genotype CC, 2 genotype TT, 3 genotype TC

**Figure 2:**
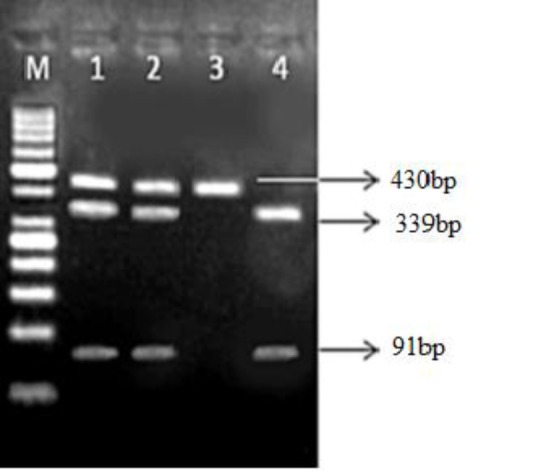
The digestion pattern of Afe1 restriction enzyme on 2% gel agarose at -56 SNP. M :Marker 50bp, 1, 2 genotype AG, 3 genotype GG, 4 genotype AA

γTable 5). Two different patterns of polymorphisms were analyzed by RFLP in -611 (Figure 1) and -56 positions (Figure 2). Logistic regression analyses revealed that genotype frequency of -611 CC was significantly higher in patients than controls (*P *< 0.024, OR = 4, 95% CI = 1.21-13.3) and genotype frequency of -56 AA was not higher in controls than patients (*P*= 0.94, OR = 1.04, 95% CI = 0.41-2.66). Therefore, a significant association was observed between patient and control groups in -611 polymorphism positions.

**Table 4:** Clinical and other characteristics of brucellosis patients

	Case	control	X^2^	*P*
Gender				
Male Female	46(76.7) 14(23.3)	65(95.6) 3(4.4)	9.9	0.02*
Education				
**Illiterate** **Diploma** **BS** **Msc&PhD**	50(84.7) 8(13.8) 0 1(1.7)	41(60.3) 20(29.4) 5(7.4) 2(3)	10.8	0.013*
Location				
**Urban** **Rural**	15(25.4) 44(74.6)	46(67.6) 22(32.4)	22.5	<0.001*
History of brucellosis				
**Yes** **No**	5(8.5) 54(91.5)	0(0) 68(100)	6	0.014*
Dairy product				
**Yes** **No**	48(81.4) 11(18.6)	52(76.5) 16(23.5)	0.5	0.5
Domesticate				
**Yes** **No**	51(86.4) 8(13.6)	10(14.7) 58(85.3)	65.1	<0.001*

* : Statistically significant

**Table 5:** Comparison of frequencies of IFN-  gene polymorphisms between patients with brucellosis and healthy controls

Genotypes and alleles	Patients (%)	Controls* (%)*	OR	95% CI P	*P*
-611					
TT TC CC	12(19.7) 36(6.6) 12(19.7)	24(35.4) 38(55.9) 6(8.8)	1** 1.89 4	(0.83-4.34) (1.21-13.3)	0.13 0.024*
allele					
T C	71(59.1) 71(59.1)	50(36.7) 86(58.8)	1.7	(1.071-2.78)	0.027*
-56					
AA AG GG	20(32.4) 26(43.7) 14(23.9)	22(32.4) 30(44.1) 16(23.5)	1.04 0.99 1**	(0.41-2.66) (0.41-2.41)	0.94 0.98
allele					
A G	77(64.1) 65(54.1)	76(55.8) 62(45.5)	0.97	(0.6-1.55)	0.89

* : Statistically significant

** Reference group

## Discussion

Genetic diversity in the host population, including SNPs and the genes involved in immune response, particularly cytokines, are very important in developing various diseases such as cancer (12) inflammatory and autoimmune diseases (13) as well as chronic infections (14). Cytokines are essential in the regulation of immune response. Polymorphic gene sequences of cytokines can be potentially susceptible to various infectious diseases in humans (15). Therefore, it could be concluded that susceptibility to brucellosis might result from genetic variations in the immune system of the host against *Brucella* infection in different populations. Rapid developments in the human genome provides great opportunities to relate genetic variations to the risks of diseases. INF-γ is a cytokine which is vital in regulating the immune response and defense against viruses and intracellular pathogens. It is known as a glycoprotein affecting immunological polytrophic (16).

Thus, gene polymorphisms of cytokines and their receptors are attractive candidates as genetic factors in immune-mediated diseases, and have been reported to be associated with susceptibility to some inflammatory and infectious diseases (17).

This research attempted to determine the potential relationships between cytokine gene receptor polymorphisms and brucellosis. 128 adults, including 60 patients and 68 healthy controls, were examined. Polymorphisms in IFN-γ R1 receptor gene promoter were analyzed in both patients and controls. The Polymorphisms of IFN-γ R1 gene promoter were identified, including one SNP at -611 position. The results indicated that the frequency of CC genotype was highly significant in patients compared to controls at -611 position, while the frequency of TT genotype was highly significant in controls compared to patients. 

The results of the current study suggested that carriers of mutant allele C at position -611 acts as risk factors for chronic brucellosis in Iranian subjects.

The patients who inherited a high prevalence of C allele and CC genotype were more susceptible to infected brucellosis, which is more likely to develop the disease. According to the previous studies, IFN-γ-induced T-cell responses are very important in promoting immunity to intracellular *B. abortus *(18) and controlling brucellosis in humans and animals (19). Although a relationship has been reported among SNPs in other cytokines and brucellosis, it has been shown that the inheritance of 590CC genotype in IL-4 (20) and -137G, +113T, +127C, and codon 35/3A alleles in IL-18 are associated with resistance (21). Whereas carrying +874 AA genotype in IFN-γ, producing high/medium IL-10 genotypes, and producing high IL-6 genotype are associated with susceptibility to brucellosis (22). 

Zhou et al. in China demonstrated that IFN- 611 and -56 C/T SNP in the IFNγ promoters are markers of brucellosis infection (19). Bravo et al. reported that IFNγ AA genotype was found in patients with brucellosis, with a significantly high rate compared to controls (23). In another study, Skandarinasab et al. showed that IFNγ AA genotype and A allele are risk factors of brucellosis infection in the Iranian population (24).

The results of this study showed that T allele is more frequent in controls than in patients at position -611. The presence of this allele may protect healthy people against *Brucella *infection. Unlike this study, Kardom et al. detected two SNPs positioned at the promoter region of IFNγ R1 in patients with tuberculosis (25). Their results showed that -56 T/C SNPs were related to chronic infection. Moreover, polymorphisms were studied within the IFNγ R1 gene promoter in a Chinese HBV infected population. The results indicated that the mutant T allele was associated with susceptibility to chronic HBV infection. However, there was no polymorphism at position -611 (19). In the current study, there was no significant correlation in IFNγ R1 gene promoter at position -56 between patients with brucellosis and controls. In a similar study, Hedayatizadeh et al. found that there is no significant relationship between allelic and genotype frequencies of G5644A polymorphism of IFNγ in brucellosis patients and controls. They reported a high frequency of the 5644A allele in patients with focal brucellosis (26). Besides, genotypes and alleles, the inherited combination of SNPs and polymorphism could influence susceptibility to many diseases. Several other studies have been performed on IFNγ polymorphism at the -611 position and infectious diseases, including pulmonary tuberculosis, HIV1/AIDS infection, retinochoroiditis, toxoplasmosis, and chronic hepatitis C virus infection (27).

In this study, AST, ALT, and ALP serum levels were measured in brucellosis patients. According to the results, there was no correlation between these enzymes and brucellosis. Boshy evaluated AST and ALT serum levels and showed that the level of these enzymes were significant in patients with infected brucellosis compared to controls (*P*<0.05) (28), which represented hepatocellular damage. Moreover, the results indicated that the number of males infected with brucellosis was more than that of females. The higher incidence rate of brucellosis in males might be attributed to the fact that men deal more with domestic animals and their products in rural areas. The results of this study showed that brucellosis was more common in rural areas than in urban areas. This can be attributed to the consumption of infected cheese and milk products. Many studies have reported that the consumption of unpasteurized cheese and milk is a significant risk factor of brucellosis (29). The present study indicated that there is a significant relationship between educational levels and brucellosis infection. Illiterate individuals were among the groups with high rate of infection because knowledge about the transmission routes of brucellosis can protect them against the infection. Also, direct exposure is one of the most significant transmission routes of the disease (30).

## Conclusion

These results should be tested in a larger group of patients in order to develop treatment and vaccination strategies to achieve a better understanding of *Brucella *γ C allele with brucellosis in the studied population. According to the results, C allele, particularly at position -611 within IFNγ R1 gene promoter was related to a high risk of disease and can be considered as a risk factor for the susceptibility to brucellosis.
